# MiR-1254 suppresses the proliferation and invasion of cervical cancer cells by modulating CD36

**DOI:** 10.1186/s12967-022-03582-6

**Published:** 2022-08-26

**Authors:** Jun Zhang, Xing Li, Jing Yang, Yan Zhang

**Affiliations:** Department of Obstetrics and Gynecology, Renmin Hospital of Wuhan University, Wuhan, 430060 Hubei China

**Keywords:** Cervical cancer, miR-1254, CD36, Proliferation, Invasion

## Abstract

**Background:**

This study aimed to elucidate the roles of miR-1254 in cervical cancer progression and to explore the underlying mechanisms.

**Methods:**

The expression levels of miR-1254 in normal-cancer cervical tissues and cells were measured using quantitive real-time polymerase chain reaction (qRT-PCR). The invasive and proliferative abilities of cervical cancer cell lines transfected with negative control (NC) mimic or miR-1254 mimic were measured using transwell, CCK-8, and colony formation assays. The binding sites between CD36 and miR-1254 were determined using luciferase reporter assays. The correlation of CD36 and miR-1254 with cervical cancer development was re-confirmed by co-transfection of miR-1254 mimic and CD36 overexpression using CCK-8, colony formation, transwell and western blot assays.

**Results:**

MiR-1254 was expressed at significantly lower levels in the cervical cancer cell lines and tissues than in the controls. The functional assays revealed that upregulation of miR-1254 inhibited the invasion and proliferation of cervical cancer cells. The luciferase reporter assays demonstrated that CD36 messenger RNA and miR-1254 bound to one another. CD36 overexpression reversed the inhibitory effects of upregulated miR-1254 in the cervical cancer cells, suggesting that miR-1254 regulates cervical cancer progression by modulating CD36.

**Conclusion:**

miR-1254 attenuated the invasion and proliferation of cervical cancer cells by modulating the expression levels of CD36.

## Background

Cervical cancer is the fourth most commonly diagnosed cancer and gynaecological cause of cancer-related deaths worldwide [1]. Several studies have found that HPV infection exerts important effects on the development of cervical cancer, which causes abnormal expression of numerous tumour-associated genes and microRNAs (miRNAs) [2, 3]. Integrating surgery and adjuvant chemotherapy are the preferred methods for treating cervical cancer; however, high rates of occurrence and metastasis lead to poor prognosis [4]. These findings suggest that it is necessary to further explore the molecular mechanisms of cervical cancer progression to discover novel therapeutic targets to block the development of cervical cancer.

Over the past few decades, increasing attention has been paid to the role of miRNAs in the onset and development of cervical cancer [5]. MiRNAs are a type of non-coding RNAs that contain fewer than 22 nucleotides and many RNA-binding sites for various target genes. They can regulate the expression levels of target messenger RNAs (mRNAs) to exert modulatory effects on the development of many tumours, including cervical cancer [6]. For example, upregulation of miR-1258 suppresses the invasion, migration and proliferation of cervical cancer cells by binding to the 3′UTR of E2F1 mRNA to promote apoptosis [7]. MiR-489 plays an anti-tumour role in cervical cancer by inactivating PI3K and AKT and upregulating the expression levels of P53 [8]. Similarly, miR-1254 is also well understood, and several studies have found that it acts as an inhibitor in some malignant tumours. MiR-1254 overexpression has been reported to inhibit the invasion, migration, proliferation, and epithelial–mesenchymal transition (EMT) of gastric cancer cells by increasing the expression of Smurf1 and inactivating the PI3K/AKT signalling pathway [9]. In addition, upregulation of miR-1254 also attenuated the migration ability of colon cancer cells by targeting PSMD10 [10]. The study findings suggest that miR-1254 is an important regulator of cancer development. In cervical cancer, Zhou et al. found that miR-1254 was significantly downregulated in cervical cancer after comparing its expression levels among 181 paired cancer-normal cervical tissues using quantitative real-time polymerase chain reaction (qRT-PCR) [11]. Nevertheless, studies on the effects and specific mechanisms of miR-1254 in cervical cancer development are scarce. These results stimulated our interest in exploring the specific effects of miR-1254 in cervical cancer. Thus, we performed this study to elucidate the effects of miR-1254 on cervical cancer progression, to analyse the underlying regulatory mechanisms and to explore new targets to block cervical cancer development.

## Methods

### Clinical specimen collection and processing

A total of 30 paired cancer-normal cervical tissues, obtained during surgery, were collected from patients, and the diagnosis was confirmed via postoperative pathology. No patients had been treated with radiotherapy or chemotherapy prior to surgery. The patients were divided into two groups according to the WHO criteria for the classification of cervical cancer, including 15 patients classified under stage I–II and 15 patients classified under stage III–IV. After surgical resection, all the tissues were stored in liquid nitrogen. Before collection of these samples, study approve was obtained by the Ethics Review Board of Renmin Hospital of Wuhan University, and written informed consent from all patients enrolled in this study.

### Cell culture

The normal cervical cell line (Ect1/E6E7), cancerous cell lines (HeLa, C33a, SiHa and CaSki) and 293T cells were obtained from the American Type Culture Collection. The cells were then incubated in DMEM containing 10% foetal bovine serum (FBS, Invitrogen, Carlsbad, CA, USA) and 1% penicillin/streptomycin (Invitrogen), and cultured at 37 °C with 5% CO_2_.

### RNA extraction and qRT-PCR

Total RNA was extracted from the cells and tissues using a TRIzol reagent (Invitrogen) according to the manufacturer’s instructions. cDNA was synthesised using a reverse transcription kit (Takara, Dalian, China). Subsequently, the expression levels of miRNAs and CD36 were measured using qRT-PCR with SYBR II Premix Taq (Takara). GAPDH was used as an internal control and the fold-expression levels of miR-1254 and CD36 were calculated using the 2-ΔΔCT method. All experiments were carried out in triplicates. The miR-1254 primer was obtained from Sangon Biotech (Shanghai, China). The primers of relevant genes used were as follows: miR‐1254 forward primer: 5′‐AGCCTGGAAGCTGGAGCCTGCAGT‐3′; miR-1254 reverse primer: 5′‐GCGAGCACAGAATTAATACGAC‐3′; CD36 forward primer: 5′-TGTGCAAAATCCACAGGAAG-3′; CD36 reverse primer: 5′-GCCACAGCCA GATTGAGAAC-3′. GAPDH forward: 5′-CTGGGCTACACTGAGCACC-3′, GAPDH reverse: 5′-AAGTGGTCGTTGAGGGCAATG-3′.

### Plasmid construction and transfection

MiR-1254 mimics (GenePharma, Hangzhou, China) were used to upregulate the expression levels of miR-1254, while miR-1254 inhibitors (GenePharma) were transfected into the cells to reduce miR-1254 expression. Upregulation of CD36 in the target cervical cancer cells was performed using pIRES2-ZsGreen1-CD36 (Hanbio, Shanghai, China) and labelled CD36-ov. Empty pIRES2-ZsGreen1 plasmid was also used as a control (CD36-ctrl). SiHa and CaSki cervical cancer cells were transferred into 6-well plates at a density of 1 × 10^5^ cells per well. Then, the target cells were transfected with miR-1254 or miR-NC mimic, miR-1254 inhibitor, NC inhibitor, CD36-ov, or CD36-ctrl using Lipofectamine 3000 (Invitrogen) according to the manufacturer’s instructions.

### Cell proliferation assay

The SiHa and CaSki cervical cancer cells transfected with miR-1254 mimic or NC mimic and CD36-ov or CD36-ctrl were transferred into 96-well plates at 1 × 10^3^ cells per well. After incubation for 4, 24, 48, 72, and 96 h, the OD value of the transfected cells at 450 nm was detected by CCK-8 (Dojindo, Kumamoto, Japan) using a microplate reader. The experiments were performed in triplicates.

### Colony-formation assay

The transfected SiHa and CaSki cervical cancer cells were transferred into 6-well plates at 1 × 10^3^ cells per well and then cultured in an incubator at 37 °C for 7 days. After the formation of visible colonies, the cells were stained using a Giemsa stain kit (BA-4017, BASO, China) according to the manufacturer’s protocol. Subsequently, the number of colonies was counted using an optical microscope (Olympus DX51, Olympus, Denmark).

### Luciferase reporter assay

The mutant and wild-type vectors of the pMIR-CD36-3′-UTR were purchased from GenePharma (Hangzhou, China). The SiHa and CaSki cervical cancer cells were co-transfected with miR-1254 mimic or NC mimic and pMIR-CD36-3′-UTR mutant-type vector or wild-type vector. Subsequently, the cells were cultured at 37 °C for 48 h. The luciferase activity of the target cells was measured using the dual-luciferase assay kit (Promega, Madison, WI, USA), according to the manufacturer’s instructions.

### Cell invasion and migration assays

The effects of miR-1254 on the invasion of the SiHa and CaSki cervical cancer cells were measured using transwell chambers with an 8-µm pore size (Corning, NY, USA). Briefly, the transfected SiHa and CaSki cells incubated with serum-free DMEM were seeded into the upper chamber, which had been coated with 50 µl Matrigel (BD, Beijing, China). We added 600 µl DMEM containing 10% FBS (Invitrogen) to the lower chamber. After 24 h of culture, the non-invading SiHa and CaSki cells were removed, and the invading cells were fixed with 4% paraformaldehyde and stained with haematoxylin–eosin. The number of invaded cells was counted using an optical microscope (Olympus DX51).

### Western blot

Protein samples of the SiHa and CaSki cells co-transfected with miR-1254 and CD36-ov or CD36-ctrl were extracted in radio immunoprecipitation assay lysis buffer (KeyGen BioTech, Nanjing, China). Subsequently, they were quantified using a bicinchoninic acid kit (KeyGen BioTech, Nanjing, China) according to the manufacturer’s protocol. The equal masses of proteins were separated using 12% SDS-PAGE and then transferred to PVDF membranes (Millipore, Billerica, MA, USA). The membranes were blocked using 5% non-fat milk in Tris-buffered saline with Tween-20 (TBS-T). The blots were incubated with GAPDH and CD36 antibodies overnight at 4 °C. After washing of the membranes with TBS-T for three times (10 min/wash), the membranes were incubated with secondary antibodies at 37 °C for 1 h. We repeated the washing step with TBS-T, and the protein signals were analysed using an enhanced chemiluminescence detection kit (Thermo Fisher, USA).

### Statistical analysis

Data were processed using SPSS 19.0 and expressed as means ± standard deviation. All experiments were performed at least in triplicates. Differences between the two groups were analysed using the paired Student’s t-test, and statistical significance was set at P values of < 0.05.

## Results

### Reduced miR-1254 expression was observed in the human cervical cancer tissues and cell lines

**Fig. 1:**
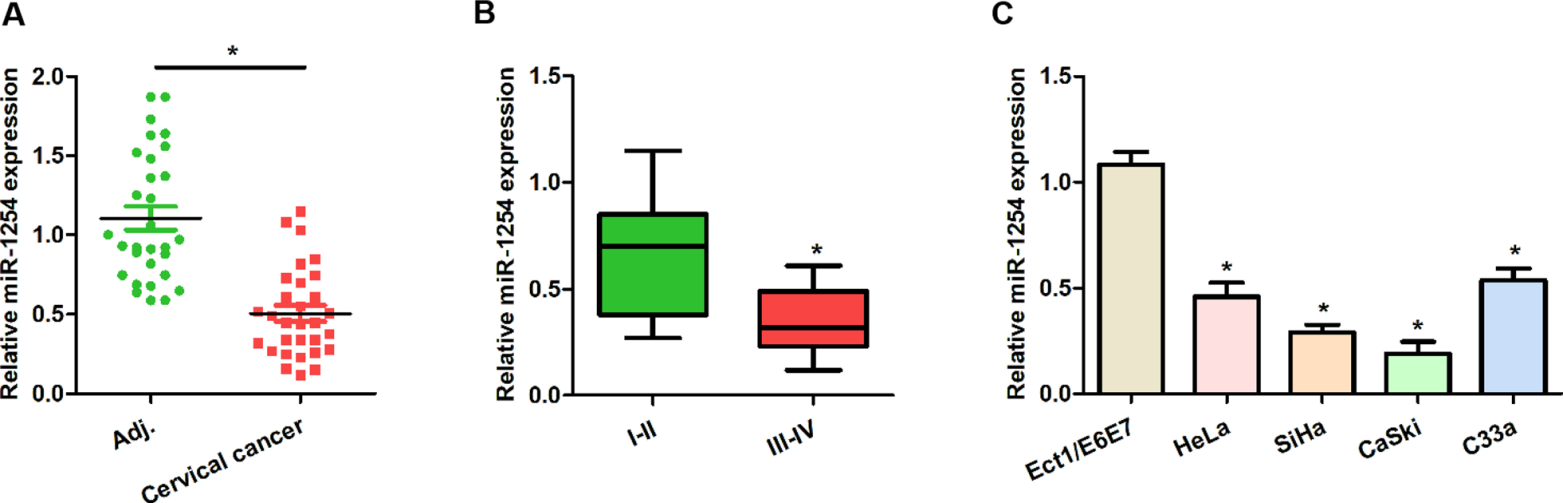
MiR-1254 levels in the cervical cancer tissues and cell lines. **A** Low miR-1254 levels were measured in the cervical cancer samples compared to those in the adjuvant tissues. **B** The miR-1254 levels were lower in the patients with cervical cancer with late pathological stages. **C** MiR-1254 levels in the cervical normal and cancer cell lines. *P < 0.05

The expression levels of miR-1254 in the 30 paired normal-cancer cervical tissues were measured using qRT-PCR. We found that the miR-1254 expression was significantly lower in the cancerous tissues than in the normal tissues (Fig. 1A). Furthermore, the expression levels of miR-1254 were lower in the patients with advanced pathological stages (Fig. 1B). These results suggest that miR-1254 is related to the progression of cervical cancer and may indicate a poor prognosis. In addition, the expression levels of miR-1254 in the normal cervical cell lines (Ect1/E6E7) and cancerous cell lines (HeLa, SiHa, CaSki and C33a) were also examined, and a significant low expression was found in the latter (Fig. 1C).

### Upregulation of miR-1254 attenuated the proliferative and invasive abilities of the cervical cancer cells

**Fig. 2:**
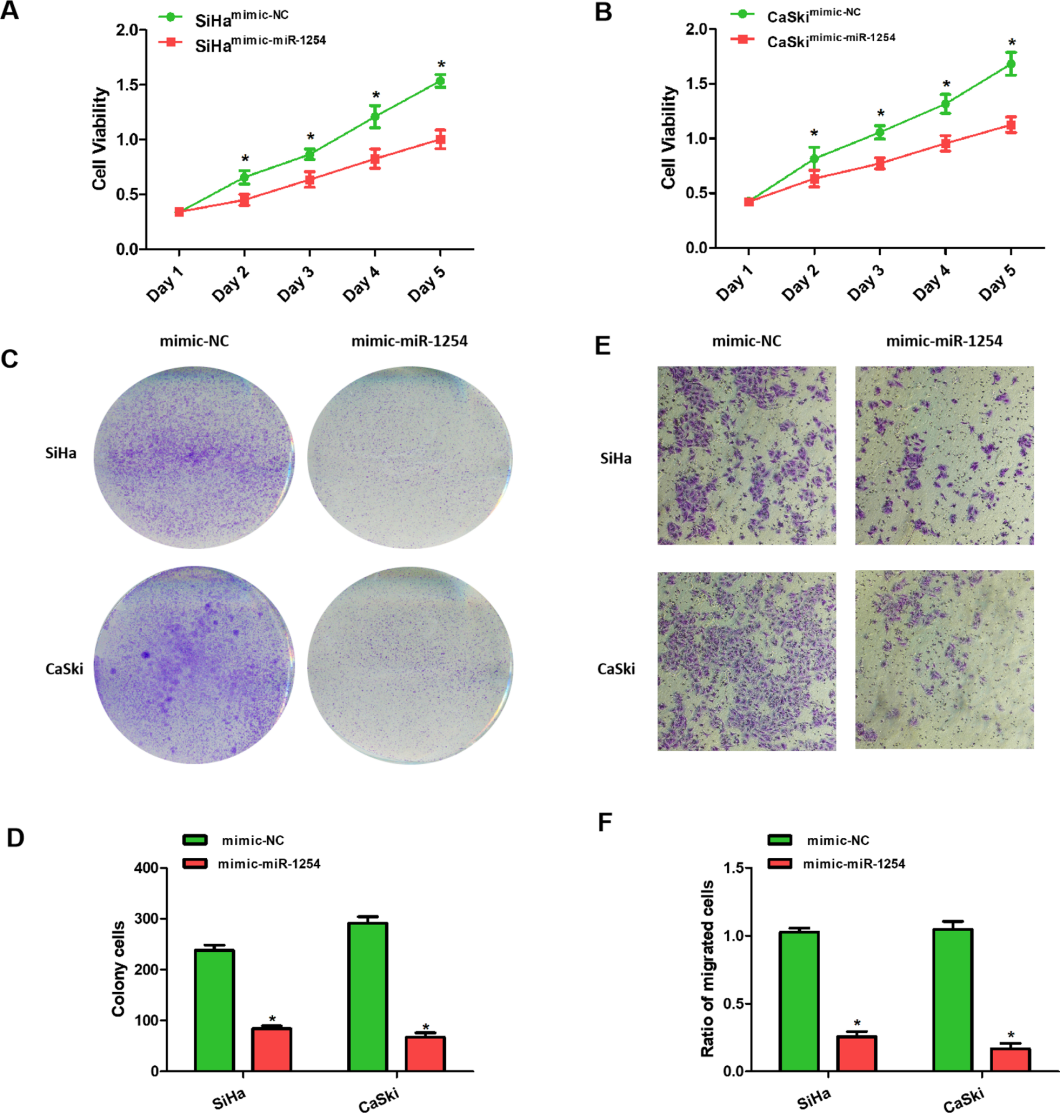
Upregulation of miR-1254 inhibited the progression of cervical cancer. **A**, **B** The CCK-8 assay showed that transfection with miR-1254 mimic inhibited the proliferation in the CaSki and SiHa cervical cancer cells. **C**, **D** The colony formation assay showed that miR-1254 overexpression significantly reduced the number of colonies. **E**, **F** Transfection with miR-1254 mimic suppressed the invasion in the CaSki and SiHa cervical cancer cells examined using a transwell assay (magnification, ×100). *P < 0.05

We transfected miR-1254 mimic or NC mimic into the SiHa and CaSki cells to construct miR-1254 overexpression cell models, because of the lower miR-1254 expression in the SiHa and CaSki cells. After transfection, an elevated miR-1254 expression level was confirmed on qRT-PCR. The CCK-8 and colony formation assays revealed that miR-1254 overexpression inhibited the proliferation of the SiHa and CaSki cervical cancer cells (Fig. 2A–D). The transwell assay demonstrated that elevated expression levels of miR-1254 suppressed the invasion of the SiHa and CaSki cervical cancer cells (Fig. 2E, F).

### MiR-1254 targeted CD36

**Fig. 3:**
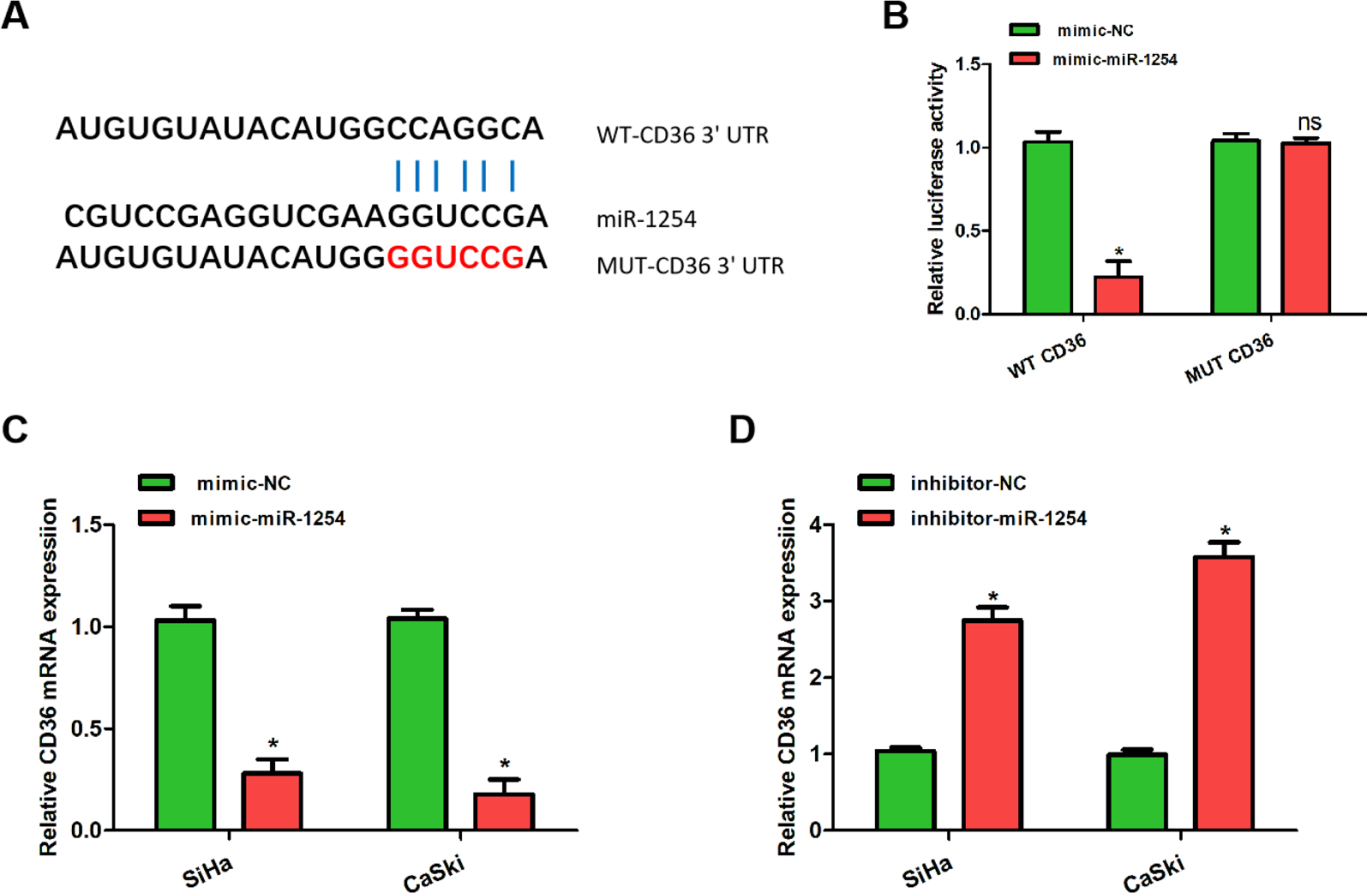
MiR-1254 targeted CD36. **A** Binding sites for miR-1254 and CD36. **B** The association between miR-1254 and CD36 was confirmed using luciferase activity assays. **C**, **D** The expression levels of CD36 mRNA were measured using quantitative real-time polymerase chain reaction after downregulation or upregulation of miR-1254 to further confirm the correlation between CD36 and miR-1254. *P < 0.05

The binding sites of miR-1254 and CD36 were reported in OSCC in a recent study [12]. Herein, we found the same relationship using the TargetScan database (http://www.targetscan.org) and confirmed this using a luciferase reporter assay (Fig. 3A, B). The luciferase activity was significantly lower in the miR-1254 mimic and wild-type CD36-3′-UTR co-transfected groups than in the mutant-type CD36-3′-UTR group (Fig. 3B). To further verify this, we measured the expression levels of CD36 in the SiHa and CaSki cells that had been transfected with miR-1254 mimic or NC mimic, miR-1254 inhibitor or NC inhibitor. We found that upregulated miR-1254 reduced CD36 expression, while downregulated miR-1254 increased CD36 expression (Fig. 3C, D). Taken together, these results suggest that CD36 is a target gene of miR-1254.

### Overexpression of CD36 rescued the effects of miR-1254 on cervical cancer cell proliferation and invasion

**Fig. 4:**
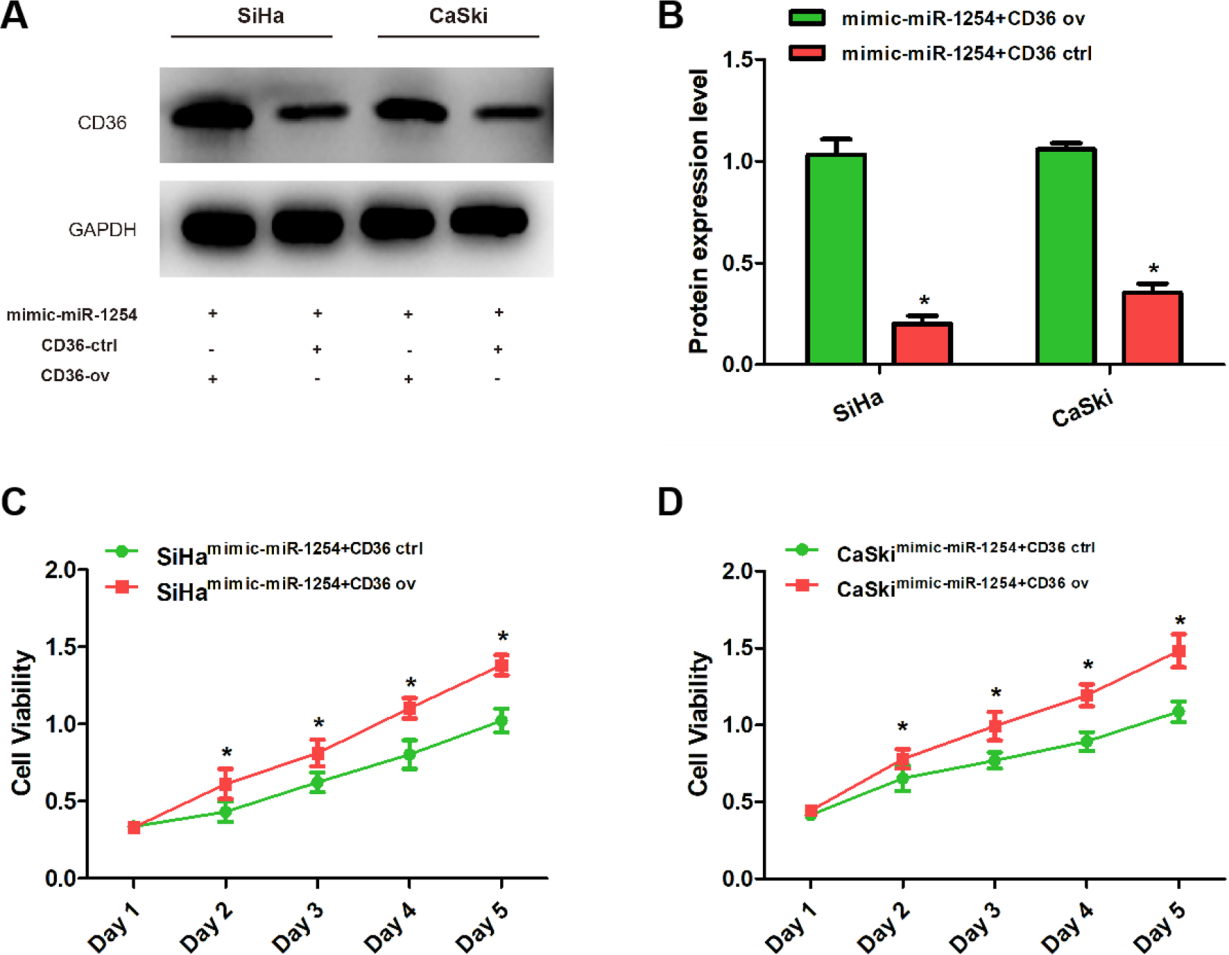
CD36 overexpression rescued the effects of miR-1254 on the cervical cancer cells. **A**, **B** Western blot and quantitative real-time polymerase chain reaction revealed that transfection of pIRES2-ZsGreen1-CD36 significantly increased the CD36 expression levels in the CaSki and SiHa cells transfected with miR-1254 mimic. **C**, **D** The CCK-8 assay revealed that upregulation of CD36 promoted the proliferative ability of the CaSki and SiHa cells transfected with miR-1254 mimic. *P < 0.05

**Fig. 5:**
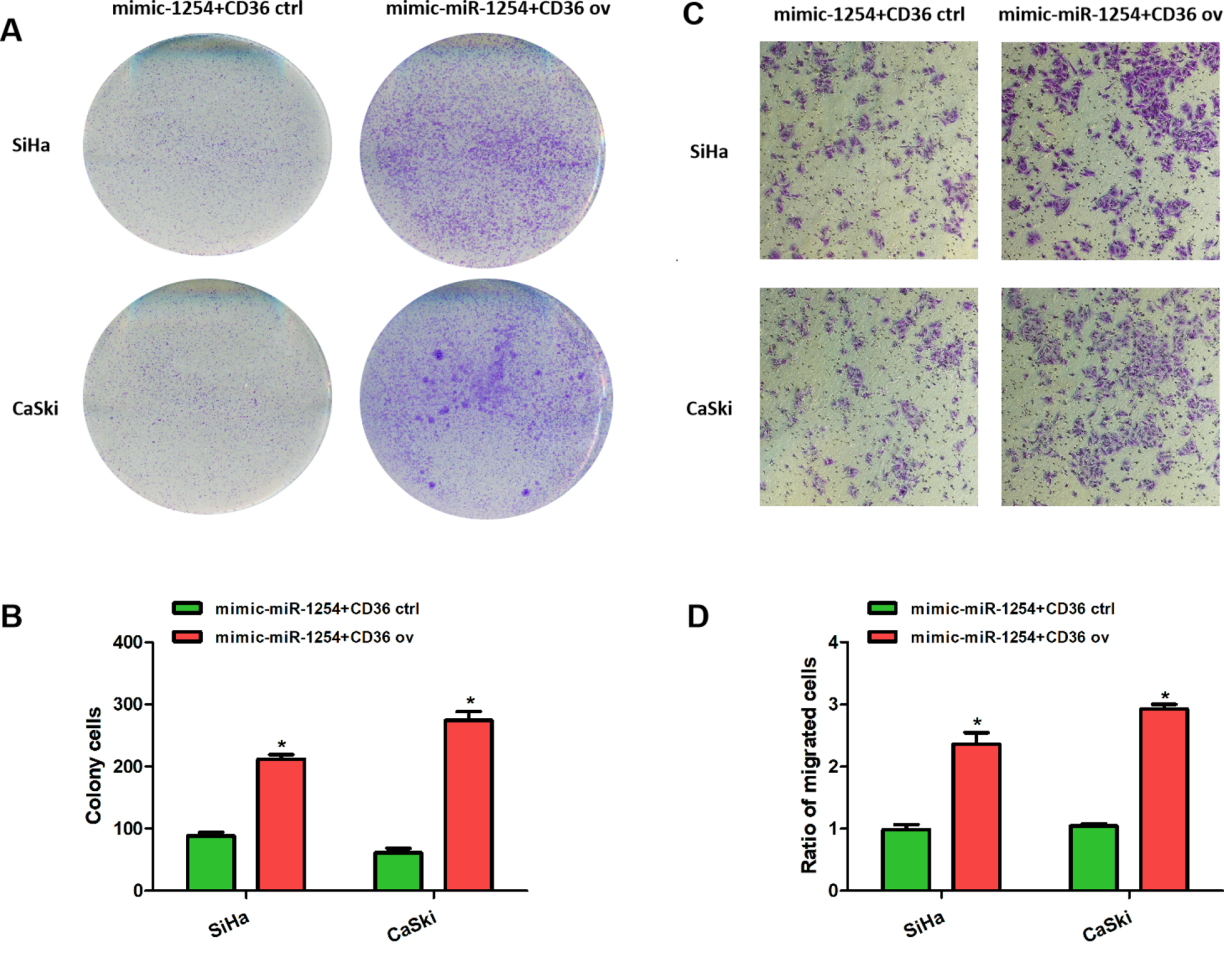
CD36 overexpression rescued the suppressive effects of miR-1254 on cervical cancer cell proliferation and invasion. **A**, **B** The colony formation assays revealed that upregulation of CD36 promoted the proliferative ability of the SiHa and CaSki cells transfected withmiR-1254 mimic. **C**, **D** The transwell assays showed that CD36 overexpression enhanced the invasive ability of the SiHa and CaSki cells transfected with miR-1254 mimic (magnification, ×100). *P < 0.05

We constructed miR-1254 and CD36 co-overexpressing SiHa and CaSki cervical cancer cells using miR-1254 mimic and pIRES2-ZsGreen1-CD36. A control group was constructed using miR-1254 mimic and empty plasmids. After that, the expression of CD36 at the protein level was examined via western blot, and a high CD36 expression was detected in the pIRES2-ZsGreen1-CD36 group (Fig. 4A, B). Subsequently, the CCK-8 and colony formation assays showed that the proliferative ability of the cervical cancer cells was significantly enhanced after the expression of CD36 increased (Figs. 4C, D, 5A, B). The transwell assays showed that upregulation of CD36 rescued the promotive effects of miR-1254 on cervical cancer cell invasion (Fig. 5C, D). These results suggest that miR-1254 suppresses cervical cancer progression by targeting CD36, and increased CD36 expression reverses the inhibitory effects of miR-1254.

## Discussion

In recent years, substantial dysregulated miRNAs with important regulatory effects have been found in cervical cancer cell lines and tissues [13–15]. MiRNAs exert effects on all biological behaviours of cervical cancer onset and development, including apoptosis, proliferation, invasion, migration, and drug resistance [16–18]. For example, miR-20b enhances the invasive ability of cervical cancer cells by upregulating EMT [19]. Wang et al. found that the proliferative and invasive abilities of cervical cancer could be enhanced by downregulation of miR-214, which was completed by modulating the PI3K/AKT/mTOR signalling pathway or targeting ARL2, EZH2, and FOXM1 [20–23]. MiR-145 suppresses the proliferation and metastasis of cervical cancer cells by inhibiting the Wnt/β-catenin pathway [24].

Among these miRNAs, miR-1254 is well known. Dysregulation of miR-1254 has crucial effects on cancer onset and development. Some studies have reported that miR-1254 plays an important anti-tumour role in various cancers [9, 10]. For example, its downregulation was examined in OSCC cells, and functional experiments showed that miR-1254 overexpression inhibited the metastasis of OSCC by modulating the MAPK signalling pathway [25]. However, some studies have identified the oncogenic effects of miR-1254 [12, 26]. However, to date, no reports have revealed the specific role and mechanism of miR-1254 during the biological progression of cervical cancer up to date. In this present study, we explored the specific role of miR-1254 in cervical cancer development and analysed its underlying mechanisms. Significant downregulation of miR-1254 was observed in both cervical cancer cell lines and tissues, suggesting a negative association between miR-1254 expression and cervical cancer progression. Further functional experiments suggested that increasing miR-1254 levels suppressed the invasion and proliferation of cervical cancer cells. The role of miR-1254 in regulating cervical cancer and its potential molecular mechanism might provide a novel direction for the study of cervical cancer occurrence and development.

Several lines of evidence suggest that CD36, a receptor protein, is related to the onset and progression of tumours and normally acts as a cancer promoter. For example, Pascual et al. found that blocking CD36 inhibited the metastasis of human oral cancer in mouse models [27]. CD36 also plays a scavenger receptor role and regulates cancer stem characteristics in glioblastoma [28]. Zhao et al. found that CD36 regulates cell cycle arrest, apoptosis and migration of breast cancer cells by acting as a fatty acid translocase (FAT) [29]. By acting synergistically with TGF-β, CD36 enhances EMT and promotes metastasis in cervical cancer cells [30]. These lines of evidence suggest that CD36 may be a potential therapeutic target for the treatment of various cancers, including cervical cancer. Additionally, data from the TargetScan database (http://www.targetscan.org) showed that CD36 is a direct target of miR-1254. Thus, we wondered whether miR-1254 could affect CD36 expression in cervical cancer cells. The luciferase reporter assays confirmed the binding sites between CD36 and miR-1254. We constructed miR-1254 and CD36 co-overexpressing cervical cancer cells and performed CCK-8, colony formation, and transwell experiments. The analyses showed that increased CD36 overexpression reversed the suppressive effects of miR-1254 mimic on cervical cancer cell proliferation and invasion compared with miR-1254 mimic alone. Therefore, miR-1254 exerts anti-tumour effects in cervical cancer by modulating CD36 expression.

This study has several limitations. Firstly, the number of cervical cancer and normal tissues was insufficient. More clinical tissues should be used to confirm the expression levels of miR-1254 in cervical cancer tissues. Secondly, the effects of decreased miR-1254 levels on cervical cancer progression should be investigated subsequently. Thirdly, the expression levels of miR-1254 in blood samples should be detected to explore its potential as a biological marker for diagnosis and treatment.

## Conclusions

Taken together, the miR-1254 levels were lower in the cervical cancer tissues and cell lines, and miR-1254 exerted anti-tumour effects on cervical cancer progression. Upregulation of miR-1254 inhibited the invasion and proliferation of the SiHa and CaSki cervical cancer cells. Increased expression of CD36 rescued the effects of miR-1254 upregulation on cervical cancer progression, suggesting that miR-1254 suppresses cervical cancer development by modulating CD36.

## Data Availability

The data that support the findings of this study are available from the corresponding author upon reasonable request.

## Abbreviations

qRT-PCR: Quantitive real-time polymerase chain reaction
CCK-8: Cell counting kit-8
NC: Negative control
miRNAs: MicroRNAs
mRNAs: Messenger RNAs
EMT: Epithelial–mesenchymal transition
FBS: Foetal bovine serum
FAT: Fatty acid translocase
